# Diversity of enterobacterales in sediments of lagoons with fish farming activity and analysis of antibiotic resistance

**DOI:** 10.1016/j.toxrep.2023.02.002

**Published:** 2023-02-11

**Authors:** María Custodio, Richard Peñaloza, Alberto Ordinola-Zapata, Tessy Peralta-Ortiz, Héctor Sánches-Suárez, Enedia Vieyra-Peña, Heidi De la Cruz, Juan Alvarado-Ibáñez

**Affiliations:** a Universidad Nacional del Centro del Perú, Facultad de Medicina Humana, Huancayo, Peru; b Universidad Nacional de Tumbes, Tumbes, Peru; c Universidad Nacional Intercultural “Fabiola Salazar Leguía” de Bagua, Bagua, Peru

**Keywords:** Enterobacterales, Diversity, Lagoon sediment, Antibiotics, resistance, Fish production

## Abstract

The use of antibiotics in fish production can induce bacterial populations to develop resistance to multiple antibiotics and transfer antibiotic resistance genes to other bacteria, including clinically relevant bacteria. This study evaluated the diversity of Enterobacterales in sediment from lagoons with fish farming activity and analyzed antibiotic resistance in the central region of Peru. Sediment samples were collected from four fish-active ponds and transported to the laboratory for analysis. Bacterial diversity was analyzed using DNA sequencing and antibiotic resistance was tested using the disk diffusion method. The results showed variability of bacterial diversity in the ponds with fish farming activity. Simpson's index indicated that the Habascocha lagoon is the most diverse in bacterial species of the order Enterobacterales (0.8), but the least dominant. The Shannon-Wiener index revealed that it is the most diverse (2.93) and the Margalef index revealed that species richness in this lagoon is high (5.72). Similarity percentage analysis (SIMPER) allowed the identification of the main Enterobacterales with the highest percentage contribution in the frequencies of individuals. In general, the Enterobacterales species isolated showed multi-resistance to the antibiotics used and *Escherichia coli* was the most resistant.

## Highlights

• Abundance of *Plautia stali* symbiont was lower in lake sediment with higher abundance of *Escherichia coli*.
• *E. coli* was the pathogen with the highest percentage contribution to Enterobacterales order.
• *E. coli* and *Enterobacter* sp. isolates from fish farming lagoons showed resistance to seven or more antibiotics.

## Graphical Abstract

**Figure:**
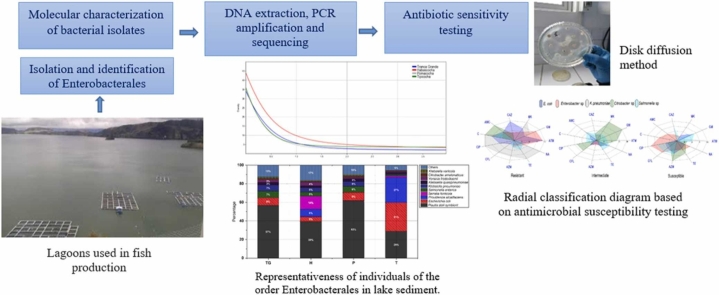

## 1 Introduction

The quality of the aquatic environment has been undergoing changes due to the contribution of a range of pollutants from different sources [3], [7]. In recent years, the aquaculture sector has shown a growth trend in its production worldwide. According to the Food and Agriculture Organization of the United Nations 2020, aquaculture increased by 3.1% annually during 1961–2017 and exceeded the annual growth of the world population (1.6%) [41]. Fish production in ponds or cages in natural water bodies accumulates organogenic sediments continuously due to intensive feeding and fecal waste [69]. Under these conditions, these production media are often exposed to infectious agents that spread rapidly through the aqueous medium [25]. This situation leads to the direct use of prophylactic and therapeutic drugs to maintain the health of aquaculture specimens.

Fish production in high Andean lagoons is often conducted in cage systems, which allows free transfer of water from the cages to the surrounding water and sediment. The use of antibiotics to treat fish infections can cause the water and sediment to become reservoirs for antibiotic resistance genes [42], [52], [72], [75], [77]. The use of antibiotics in fish production can induce bacterial populations to develop resistance mechanisms to multiple antibiotics [2], [56], e.g. enzyme production, overexpression of the ejector pump, porin modification [15] and biofilm formation that reduce susceptibility to antibiotic activity [19]. Therefore, the use of antibiotics in fish production is of growing concern as a critical point for the enrichment and dissemination of antibiotic resistance genes through mobile genetic elements [86], [1]. Horizontal transfer of antibiotic resistance genes can occur and reach other bacteria and, in some cases, even human pathogens [78]. Therefore, bacterial genomes are constantly changing, and any segment of DNA in a large bacterial population could have the opportunity for horizontal transfer [74]. Many antibiotics used in human medicine are also used in aquaculture.

The presence of antibiotic residues and antibiotic resistance genes in the aquatic environment can affect the composition of the bacterial community that plays an important role in the ecosystem [24] and endangering water quality and human health [39]. Plasmids harboring resistance determinants to various antimicrobials are transferable from pathogenic bacteria of fish and aquatic bacteria to pathogenic bacteria such as *Escherichia coli* [60], [34], *Vibrio parahaemolyticus, V. cholerae, Shigella* and *Salmonella* [73]. The spread of antibiotic resistance among diverse bacterial populations is a major clinical problem that complicates the medical use of antibiotics. Currently, extended-spectrum β-lactamases (ESBL) and carbapenemases are the most common β-lactamases detected in enterobacteria, particularly in *Escherichia coli* and *Klebsiella* pneumoniae [71]. These enzymes endow bacteria with various resistances to most advanced generation antibiotics such as β-lactams, including penicillins, cephalosporins and aztreonam [51], but no cephamycins nor carbapenems [61]. Therefore, carbapenems have been used as the treatment of choice for infections due to ESBL-producing Enterobacteriaceae, resulting in the emergence of carbapenemasa-producing Enterobacteriaceae [81].

Due to the risk posed by the presence of antibiotics in the environment, many countries have already introduced the obligation to monitor this type of contamination in the aquatic environment. For example, the European Union Commission has established a watch list of substances for Union-wide monitoring in the field of water policy. Three antibiotics (erythromycin, clarithromycin and azithromycin) were included in the first watch list and are checked and reviewed every two years. In Peru, several clinical studies have been conducted on antibiotic resistance [23], [46], but studies of resistance to these antibacterial agents in the environment are limited [67], particularly in aquatic environments. Studies of antibiotic resistant bacterial diversity using DNA sequencing in continental lake environments with fish farming activity in the central region of Peru do not exist. Most studies are focused on evaluating the trophic status of these environments and others on monitoring heavy metals. Consequently, knowing the antibiotic resistant bacterial diversity in lake environments with intensive fish farming activity can help to understand the antibiotic consumption pattern and impact of intensive fish farming and contribute to address the epidemiology of antibiotic resistance. Therefore, the importance of this study, since the central region of Peru is one of the main regions in fish production for both domestic and foreign markets. In addition, bacteria of the order Enterobacterales often function as important vectors in the dissemination of β-lactamase genes in natural bacterial ecosystems [71]. Therefore, this research evaluated the diversity of Enterobacterales in sediment from lagoons with fish farming activity and analyzed antibiotic resistance in the central region of Peru.

## 2 Materials and methods

### 2.1 Study area and sampling

**Fig. 1:**
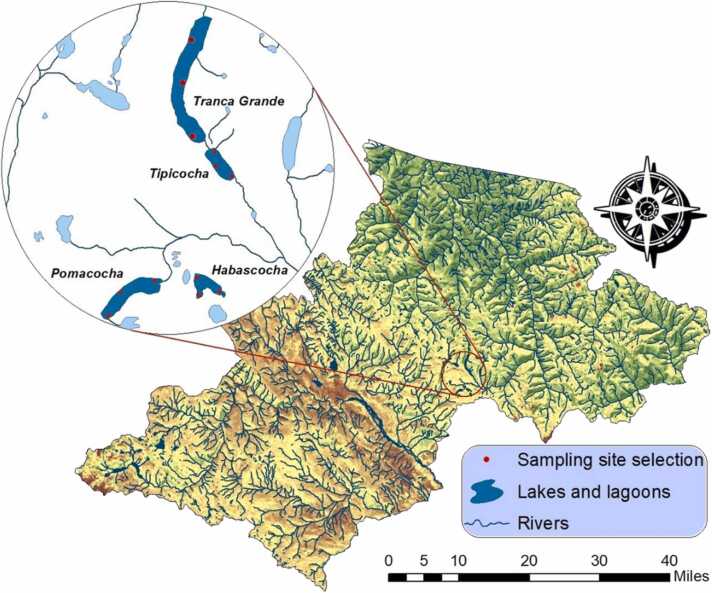
Location map of the study area in the central region of Peru.

The study area is located in the Pomacocha, Habascocha, Tipicocha and Tranca Grande lagoons of glacial origin located in the upper basin of the Perené River, in the Central Andes of Peru, at an altitude between 4310 and 4330 m above sea level [47], [48] (Fig. 1). The area and depth of these ponds vary from 80 to 164 ha and from 10 to 28 m, respectively. Water temperature varies from 9.8 to 13.1 ºC and pH from 7.7 to 8.06 (Table S1). The four lagoons are used for the intensive culture of *Oncorhynchus mykiss* (rainbow trout) in large floating cages. In 2019, the number of cages per lagoon was 122 (Tranca Grande), 35 (Tipicocha), 13 (Habascocha) and 0 (Pomacocha) (Google, 2022). Three sampling sites were established at each lagoon and at each site 250 g of surface sediment (10 cm) was collected in triplicate in November 2020. A total of 36 sediment samples were collected from the study area. Sediment samples from each lagoon were conditioned in airtight plastic bags and transported in cold chain to the Molecular Biology Laboratory of the Universidad Nacional de Tumbes for analysis [49].

### 2.2 Isolation and identification of Enterobacterales

Isolation of Enterobacterales was performed by mixing 1 g of sediment in 99 ml of sterile distilled water in a 250 ml Erlenmeyer flask, followed by shaking at 150 rpm for one hour at room temperature. Bacterial isolation was carried out from the supernatant by successive dilution methods and biochemical tests on Triple Sugar, Iron Agar (TSI) [8]. Dilutions of 10^—1^ to 10^—5^ were prepared and seeding of each dilution was performed on nutrient agar plates by surface streaking and incubation was performed at 37 °C for 24 h. Biochemical identification of individual colonies was performed by subculturing on TSI agar and molecular characterization using DNA sequencing.

### 2.3 Antibiotic sensitivity testing

Antibiotic sensitivity of bacterial isolates was determined by the disk diffusion method. Inocula of morphologically similar colonies were prepared in Tryptic Soy Broth and incubated at 35 °C until a turbidity equal to 0.5 McFarland (equivalent to 2 ×10^8^ cells/ml) was obtained by UV/VIS spectrophotometer reading at 625 nm. Sterile swabs were then dipped into the inocula and spread on Mueller-Hinton agar plates (BD DifcoTM, USA). Antibiotic discs were placed on each of the plates equidistantly at 30 mm, and the plates were incubated at 35 °C for 24 h. After incubation, the diameters of the inhibition zones were measured and the bacterial isolates were classified as "S" (susceptible), "I" (intermediate) or "R" (resistant), according to the standards of the National Committee for Clinical Laboratory Standards [35]. The *Escherichia coli* ATCC 25922 strain was used as a control for the antibiotic sensitivity test due to its tolerance to different temperature ranges and its standardization for the determination of antimicrobial susceptibility of bacteria in the aquatic environment [50]. The antibiotic discs Aztreonam (ATM), Gentamicin (GM), Amikacin (MK), Ceftazidime (CAZ), Amoxicillin - Clavulanic Acid (AMC), Chloramphenicol (C), Ciprofloxacin (CIP), Cephalexin (CFL), Azithromycin (AZM), Tetracycline (TE) and Nalidixic Acid (NA) were purchased from Biodisc SAC.

### 2.4 Molecular characterization of bacterial isolates

#### 2.4.1 DNA extraction

A set of colonies were randomly picked from each plate, cultured in brain heart infusion broth and incubated at 30 ºC, 150 rpm for 24 h. Bacterial genomic DNA extraction was performed using the PrestoTM Soil DNA Extraction Kit, following the manufacturer's instructions. Bacterial lysis was performed from 600 µl of the bacterial isolate and 750 µl of lysis buffer in a 2 ml tube with ceramic beads. Inhibitor removal was performed by transferring 600 µl of the supernatant from the tube to the silica column. The DNA was washed successively with SL3 buffer (one wash) and Wash buffer (two washes), with each wash step centrifuged at 16,000 x g for 30 s. Finally, the silica column was centrifuged at 16,000 x g for 2 min at room temperature. DNA elution was performed by applying 100 µl of elution buffer to the column, allowed to stand for 2 min and centrifuged at 16,000 x g for 2 min at room temperature. The eluted DNA was stored at − 20 °C until further analysis.

#### 2.4.2 PCR amplification and sequencing

PCR amplification was performed using Gene One and GE Healthcare Life Sciences kits by mixing 1 µl of universal 16 S rRNA F primer, 1 µl of universal 16 S rRNA R primer, 22 µl of PCR mix (containing premix buffer, MgCl2, dNTPs and taqPolymerase) and 1 µl DNA sample for a total reaction volume of 25 µl. The primers 27 F (5′-AGAGAGTTTGATCCTGGCTCAG-3′) and 1492 R (5′-GGTTACCTTGTTACGACTT-3′) were used and amplified for a product of about 1465 bp [12]. The initial concentration of both primers was 10 pMol/µl equivalent to 10 µM, with a final concentration after incorporation into the amplification mixture of 0.4 µM. Bacterial 16 S rRNA amplicon sequencing of the V3 and V4 hypervariable regions was performed using the standard Illumina MiSeq v2 next generation protocol [18], [38], [68], [21]. The construction of the library was done commercially (ADMERA HEALTH LLC, USA).

#### 2.4.3 Bioinformatic analysis of sequence reads

The raw Illumina paired reads were loaded as FASTQ files. Quality filtering was performed with the Trimmomatic v0.39 program [30] to remove primer regions, adapter regions and low quality reads (mean score < 20 and read length < 100 bp). MetaSPAdes v3.13 [59] was performed to assemble de novo DNA sequences. Amplicons were clustered in operational taxonomic units (OTU) using Kraken 2 v 2.1.2 [82] with the database SILVA v132 [66]. Data have been deposited with links to BioProject accession number PRJNA682317 in the NCBI BioProject database (https://www.ncbi.nlm.nih.gov/bioproject/).

### 2.5 Statistical analysis

The assessment of species diversity of the order Enterobacterales was performed using the number of taxa and diversity indices, such as Margalef (M) to determine species richness, Simpson (1-D) to determine dominance and Shannon-Wiener (H′) as diversity index [13], [53]. Diversity indices were considered only for the order Enterobacterales. Rényi diversity profiles were also used to classify Enterobacterales communities [28]. The diversity of the bacterial order studied was examined by rarefaction analysis to assess species richness [20]. Similarity percentage analysis (SIMPER) was used to evaluate the average percentage contribution of the individuals [14]. Analyses were calculated using PAST V4.08 software [26]. The number of species in relation to the gaps were inspected using the double clustering technique ("p" and "q") where the Euclidean dissimilarity index and the minimum variance method (Ward) were used [33]. The interpretation was based on clustering coefficients that reflect the distance at which groups merge. A large increase in the clustering coefficient suggests that two quite different clusters merged [11], for this study with a maximum distance value of 4000. The heat map was generated using R software, with the pheatmap package [36]. The antibiotic susceptibility analysis was performed with descriptive statistics (absolute frequencies) and principal component analysis with Euclidean distances with PAST software in order to determine the response of Enterobacterales to the antibiotics using the correlation ranges (0–0.1 null, 0.1–0.3 weak, 0.3–0.5 moderate and 0.5–1 strong) of the component scores for interpretation.

## 3 Results

### 3.1 Diversity of Enterobacterales in the sediment of lagoons with fish farming activity

The analysis of the diversity of species of the order Enterobacterales in lake sediment revealed variability in bacterial diversity according to lagoon (Table 1). In Habascocha, an important diversity of species was found, followed by Tipicocha, Pomacocha and Tranca Grande lagoons. However, the highest number of individuals was found in Tipicocha lagoon. The diversity indices mostly showed a positive relationship with each other according to richness or evenness. Simpson's index (1-D) indicated that the Habascocha lagoon is the most diverse in bacterial species of the order Enterobacterales (0.8), but the least dominant (0.2). This trend is due to the anthropogenic pressure associated with fish production [47], [48]. The Shannon index showed that the Habascocha lagoon is the most diverse (H′ = 2.293), while the other lagoons tend to present greater uniformity in the frequencies of species found. The Margalef Index (M = 5.719) revealed that Habascocha lagoon has a high species richness.

**Table 1:** Species diversity index for Enterobacterales order in evaluated lagoons.

Indices	Tranca Grande	Habascocha	Pomacocha	Tipicocha
Species	34	44	35	36
Individuals	1226	1842	3837	5075
Simpson (1-D)	0.6413	0.8	0.585	0.7438
Shannon-Wiener (H′)	1.766	2.293	1.576	1.7 12
Margalef (M)	4.64	5.719	4.12	4.102

**Fig. 2:**
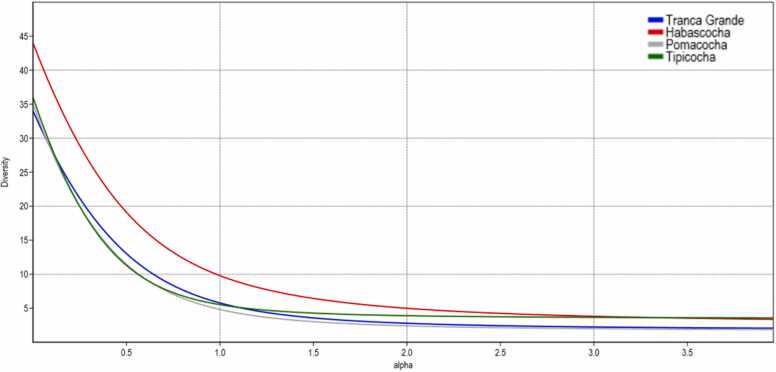
Species diversity profiles of the order Enterobacterales in lake sediments. Diversity profile using the Rényi series representing. Alpha values: a = 0 richness; a = 1 Shannon index; a = 2 inverse Simpson index (1/D).

Fig. 2 shows the diversity profiles based on the exponential of the Rényi index. This index indicates the alpha diversity of species of the order Enterobacterales. The Habascocha lagoon showed a higher number of species in the diversity profile (higher values when α = 0), denoting that it is the most diverse for the order Enterobacterales. The results also show that in this lagoon the Shannon-Wiener index (α = 1) and inverse Simpson's index (α = 2) recorded slightly higher values than the values of these indices of the other lagoons. The low richness recorded in the other lagoons reveals a low uniformity.

**Fig. 3:**
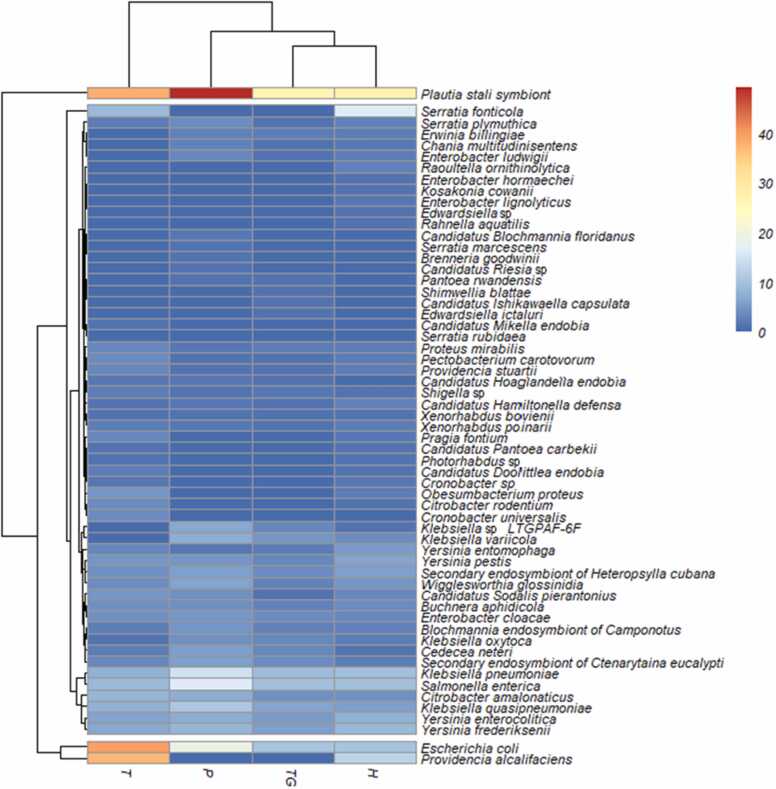
Heat map showing the cluster of Tranca Grande (TG), Habascocha (H), Pomacocha (P) and Tipicocha (T) lagoons and species of the order Enterobacterales identified by metagenomics.

Fig. 3 shows the cluster of the distribution of species of the order Enterobacterales of the studied lagoons in two groups with relevant differences. The first cluster was formed by the Tipicocha lagoon and the second group by the Pomacocha, Tranca Grande and Habascocha lagoons. The diversity indices show a greater predominance of *Plautia stali* symbiont, *Escherichia coli* and *Providencia alcalifaciens*. Tranca Grande and Habascocha lagoons have a lower dominance of the *Plautia stali* symbiont. While in Pomacocha lagoon this symbiont tends to be more dominant. This would explain the low value of the Shannon-Wiener index for this lagoon despite having a higher species frequency. *Providencia alcalifanciens* is the second most frequent species in the studied lagoons. A higher frequency of this species was found in the Tipicocha lagoon, as well as *Escherichia coli*. The second cluster was able to classify three major groups. The first group indicates the dominance of *Plautia stali* symbiont in Pomacocha lagoon. The second group determines the positive relationship between the distribution and frequency of *Escherichia coli* and *Providencia alcalafaris* species, especially in Tipicocha and Pomacocha lagoons. The third group is made up of the remaining species of the order Enterobacterales, with a dominance of *Raoultella ornithinolytica, Enterobacter hormaechei, Edwardsiella* sp.*, Rahnella aquatilis, Candidatus Blochmannia floridanus, Serratia marcescens, Brenneria goodwinii, Candidatus Riesia* sp.*, Pantoea rwandensis, Shimwellia blattae, Candidatus Ishikawaella capsulata, Edwardsiella ictaluri* and *Serratia rubidaea.*

**Fig. 4:**
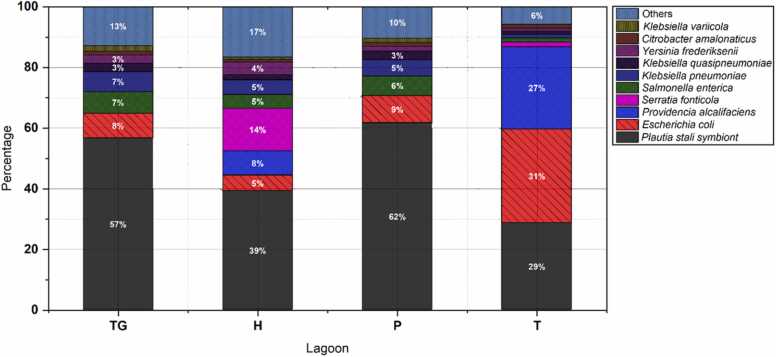
Representativeness of individuals of the order Enterobacterales in sediment from Tranca Grande (TG), Habascocha (H), Pomacocha (P) and Tipicocha (T) lagoons according to SIMPER analysis.

The SIMPER analysis allowed the identification of the main species of the order Enterobacterales that had a higher percentage contribution in the frequencies of individuals (Fig. 4). The analysis showed that the non-culturable bacteria *Plautia stali* symbiont was the most representative of the order studied, characteristic and obligatory symbiont of the insect *Plautia stali* [58]. These results revealed the presence of these or similar insects in the Peruvian Andean lagoons, especially in the Pomacocha (P) and Tranca Grande (TG) lagoons, with values of 62% and 57% contribution to the community of the order Enterobacterales, respectively. The second species of interest was *Escherichia coli*, present in all four lagoons, but more frequently in the Tipicocha lagoon (31%). Other species with an important frequency of contribution to the total number of Enterobacterales species were *Providencia alcalifaciens* (27%), characteristic of the intestinal flora of trout and salmon [6] in the Tipicocha lagoon, *Serratia fonticola* (14%), non-pathogenic species characteristic of trout skin mucosa, even beneficial for parasite control [10] in the Habascocha lagoon and the pathogenic species *Klebsiella pneumoniae* (7%) in the Tranca Grande lagoon [9].

### 3.2 Analysis of antibiotic resistance

**Fig. 5:**
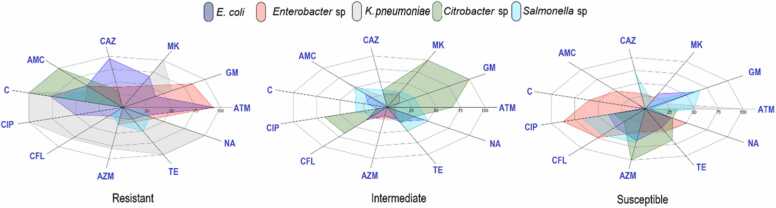
Radial classification diagram based on antimicrobial susceptibility testing (AST) differentiated by their reaction to antibiotics of species of the order Enterobacterales isolated from lake sediment. Where susceptible implies that the organism is likely to respond to treatment with the antibiotic at a standard dose, intermediate indicates that it may be effective if a higher dose can be used, and resistant implies that the organism is not likely to respond to therapy with that antibiotic.

*Escherichia coli, Klebsiella pneumoniae, Enterobacter* sp., *Citrobacter* sp. and *Salmonella* sp., were isolated and biochemically identified. Antibiotic resistance of these bacteria was tested by the disk diffusion method as described in the methods section. The percentages of isolates corresponding to susceptible, intermediate and resistant to each antibiotic are shown in Table 2. In the Pomacocha lagoon, the resistance of *E. coli* isolates (100%) was to ceftazidime and amoxicillin-clavulanic acid, those of *Enterobacter* sp. (84.62%) to aztreonam and amoxicillin-clavulanic acid and those of *Klebsiella pneumoniae* (100%) to amikacin, chloramphenicol and ciprofloxacin. All *E. coli* isolates were sensitive to gentamicin, *Enterobacter* sp., to ciprofloxacin and *Klebsiella pneumoniae* to aztreonam. In the Habascocha lagoon, the resistance of *E. coli* isolates (100%) was to aztreonam, amikacin and ceftazidime, those of *Citrobacter* sp. (100%) to amoxicillin-clavulanic acid and chloramphenicol and those of *Salmonella* sp. (66.67%) to chloramphenicol. In Tipicocha lagoon, the resistance of *E. coli* isolates (100%) was to aztreonam. The susceptibility of *E. coli* to antibiotics was very low, except for gentamicin and amikacin. In the Tranca Grande lagoon, the resistance of *E. coli* isolates (100%) was to aztreonam, gentamicin, ceftazidime and chloramphenicol and those of *Enterobacter* sp. (100%) to aztreonam and gentamicin. 100% of *E. coli* isolates were sensitive to azithromycin and 75% of *Enterobacter* sp., isolates were sensitive to amoxicillin-clavulanic acid and ciprofloxacin (Fig. 5).

**Table 2:** Antibiotic susceptibility patterns of enterobacteria isolated from lake sediment identified by biochemical analysis.

Isolation lagoon	Species/ Number of isolated	PS	Antibiotic										
			ATM	GM	MK	CAZ	AMC	C	CIP	CFL	AZM	TE	NA
Pomacocha	*Escherichia coli* (9)	S	22.22	100	44.44	0	0	11.11	0	77.78	88.89	55.56	66.67
		I	0	0	0	0	0	22.22	22.22	22.22	11.11	22.22	33.33
		R	77.78	0	55.56	100	100	66.67	77.78	0	0	22.22	0
	*Enterobacter* sp. (13)	S	0	0	23.08	23.08	15.38	69.23	100	84.62	53.85	69.23	76.92
		I	15.38	30.77	23.08	15.38	0	0	0	15.38	30.77	7.69	7.69
		R	84.62	69.23	53.85	53.85	84.62	30.77	0	0	15.38	23.08	15.38
	*Klebsiella pneumoniae* (5)	S	100	20	0	80	0	0	0	0	20	0	0
		I	0	20	0	20	60	0	0	20	0	0	0
		R	0	60	100	0	40	100	100	80	80	100	100
Habascocha	*Escherichia coli* (5)	S	0	40	0	0	40	0	100	60	60	0	0
		I	0	0	0	0	60	40	0	40	20	40	60
		R	100	60	100	100	0	60	0	0	20	60	40
	*Citrobacter* sp. (3)	S	33.33	0	0	33.33	0	0	33.33	33.33	100	66.67	33.33
		I	66.67	100	100	33.33	0	0	66.67	66.67	0	33.33	33.33
		R	0	0	0	33.33	100	100	0	0	0	0	33.33
	*Salmonella* sp. (6)	S	50	66.67	16.67	66.67	0	0	66.67	66.67	66.67	0	16.67
		I	33.33	33.33	33.33	33.33	50	33.33	33.33	16.67	0	50	50
		R	16.67	0	50	0	50	66.67	0	16.67	33.33	50	33.33
Tipicocha	*Escherichia coli* (11)	S	0	100	63.64	0	0	0	36.36	27.27	0	45.45	0
		I	0	0	9.09	18.18	36.36	18.18	0	27.27	36.36	18.18	27.27
		R	100	0	27.27	81.82	63.64	81.82	63.64	45.45	63.64	18.18	72.73
Tranca Grande	*Escherichia coli* (5)	S	0	0	20	0	0	0	20	20	100	60	60
		I	0	0	0	0	40	0	20	40	0	40	60
		R	100	100	80	100	60	100	60	40	0	0	0
	*Enterobacter* sp. (8)	S	0	0	25	37.5	75	50	75	62.5	37.5	25	25
		I	0	0	37.5	37.5	0	0	25	37.5	12.5	37.5	12.5
		R	100	100	37.5	25	25	50	0	0	50	37.5	62.5

PS: Profile susceptibility, S: Sensitive, I: Intermediate, R: Resistant. ATM: Aztreonam, GM: Gentamicin, MK: Amikacin, CAZ: Ceftazidime, AMC: Amoxicillin – Clavulanic Acid, C: Chloramphenicol, CIP: Ciprofloxacin, CFL: Cephalexin, AZM: Azithromycin, TE: Tetracycline, NA: Nalidixic Acid.

**Fig. 6:**
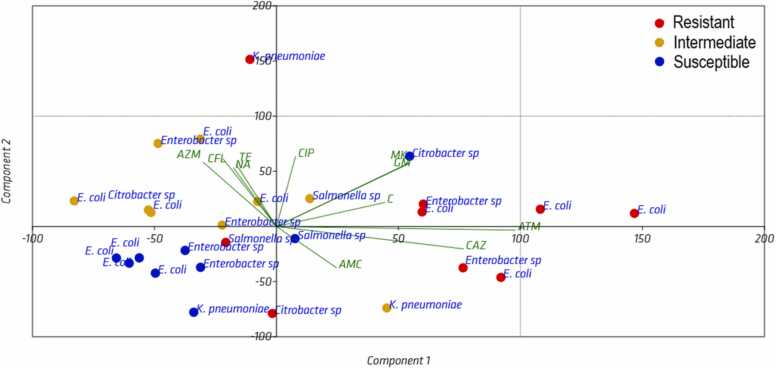
Principal component analysis of antibiotic susceptibility of species of the order Enterobacterales isolated from lake sediment. In susceptibility profile, S: Sensitive, I: Intermediate, R: Resistant. ATM: Aztreonam, GM: Gentamicin, MK: Amikacin, CAZ: Ceftazidime, AMC: Amoxicillin - Clavulanic acid, C: Chloramphenicol, CIP: Ciprofloxacin, CFL: Cephalexin, AZM: Azithromycin, TE: Tetracycline, NA: Nalidixic acid.

Fig. 6 shows the principal component analysis of antibiotic susceptibility of species of the order Enterobacterales isolated from lake sediments. The percentage of total variance of antibiotic susceptibility was 49.69% for the first two components. The first component accounted for 28.82% of the total variance and the second component for 21.5%. This low value of variance reveals the response of the distribution in bacterial resistance and sensitivity to antibiotics in the gaps of this study, as there is no equivalent efficacy for most antibiotics against a single species in the four gaps. In general, the results reveal that few antibiotics have antibacterial activity, either inhibiting growth or causing bacterial death. *E. coli* and *Enterobacter* sp., are the most resistant species to the antibiotics aztreonam and ceftazidime, indicating their stronger correspondence with the first component. The results also show that few antibiotics have antibacterial activity, either inhibiting growth or causing bacterial death. Other results show a negative correlation of azithromycin (AZM) with the first component with a correlation load value of - 0.18 (Table S2). The correlation load values of *E. coli* with respect to the first component revealed a weak correlation according to the correlation ranges (0–0.1 null, 0.1–0.3 weak, 0.3–0.5 moderate and 0.5–1 strong).

## 4 Discussion

Trout production represents 54.63% (54878 MT) of Peruvian aquaculture production and is developed in ponds and cages with average densities of 20 kg/m^3^ and 14 kg/m^3^, respectively [64]. However, the poor sanitary conditions of the ponds or cages are critical points for enrichment, growth of pathogenic microorganisms and transfer of antibiotic resistance genes [55]. The results of the bacterial diversity indexes of the order Enterobacterales found in this study reveal that the Habascocha lagoon presented the highest bacterial richness and diversity. Tipicocha was the lagoon with the lowest diversity, but the highest abundance of individuals. The diversity of Enterobacteriaceae observed in this study would be determined by the intensified use of the water body in fish farming and the loading of nutrients not consumed by the fish. Surplus food favors selected groups of bacteria that have the ability to rapidly consume these available resources or selectively eliminate certain bacteria from the bacterial community [32], [44]. The results of this study reveal the variability of bacterial diversity of the order Enterobacterales in the studied lagoons. These results are supported by Defoirdt et al. (2011) [16] who refer that bacteria in aquatic environments can reach high densities of several species, which increases the risk of contagious diseases. The analysis of the species diversity profile of Enterobacterales corroborates that Habascocha is the most diverse and even more uniform than the other three lagoons. In addition, the species distribution cluster analysis and SIMPER revealed greater dominance and percentage contribution of *P. stali* symbiont, *E. coli* and *P. alcalifaciens* with respect to the rest of the Enterobacterales species found. However, little is known about *P. stali* symbiont, a non-culturable gamma-proteobacterium species (Kobayashi et al., 2011) that has evolved from environmental bacteria [31].

The antibiotic resistance profiles recorded in the fish-use lagoons in this study are similar to resistance gradients reported by other effluent, farm and aquaculture studies [19]. Our results also reveal that lagoons used in fish production represent an important source of antibiotic-resistant bacteria. Muziasari et al. [54] support our findings and indicate that wastewater from various sources act as a reservoir of antibiotic resistant bacteria and genes. In addition, other studies report that selective pressure of antibiotic loading favors and increases the acquisition/introduction of antibiotic resistant bacteria and genes through different resistance mechanisms in the aquaculture environment, highlighting antibiotic efflux as the main mechanism of resistance in sediments [65], [83], [76]. The use of medicated feeds in fish production and the frequent use of antibiotics to control infectious diseases has led to the emergence of reservoirs of antibiotic-resistant bacteria [24]. In general, aquatic bacteria are not different from other bacteria in their responses to antibiotic exposure, and are capable of transferring antibiotic resistance genes to other bacteria [79], [63]. Consequently, antibiotic resistance genes can be exchanged between bacteria from different environments [27]. Antibiotic resistance in fish pathogens, the transfer of their genetic determinants to terrestrial animal bacteria and human pathogens, and alterations in the bacterial microbiota of the aquatic environment constitute a threat to human and animal health. The present results indicate that *E. coli* is the enterobacteria with the highest frequency and multiple resistance to ceftazidime, aztreonam, amoxicillin-clavulanic acid, chloramphenicol, ciprofloxacin and tetracycline in the studied ponds. The emergence of bacteria resistant to multiple antibiotics is a growing threat to antibiotic therapy [43], as many antibiotics used in human medicine are used in species in aquatic environments. For example, in Chile, one of the main salmon producers in America, often applies amoxicillin, erythromycin, tetracycline, and ciprofloxacin in its production process [37]. In Vietnam, Philippines, Indonesia, India and Malaysia, sulfonamides, trimethoprim, five macrolides, lincomycin and three tetracyclines have been detected in farm water [70]. In Vietnam and China, although the use of antibiotics has been reduced in the last decade, ofloxacin, enrofluxacin, sulfamethoxazole, trimethoprim and azithromycin are still detected in aquaculture products [85], [45].

Antibiotics, in and of themselves, do not cause resistance, but frequent and high exposure of antibiotics to bacteria creates selection pressure that triggers bacterial resistance mechanisms. Antibiotic resistance is a worldwide concern due to the prevalence of resistant bacteria carrying antibiotic resistance genes [80], [57]. Many studies indicate that the selection pressures imposed by antibiotics such as fluoroquinolones and the evolutionary principle of survival of the fittest have led many pathogens to evolve a variety of evasion mechanisms [17]. Resistant bacteria are increasingly difficult to treat and require less available and more toxic antibiotics [5]. 100% of *Klebsiella pneumoniae* isolates showed resistance to amikacin, chloramphenicol, ciprofloxacin and tetracycline, 80% showed resistance to cephalexin and azithromycin, 60% to gentamicin and 40% to Amoxicillin-clavulanic acid.

The increasing trend toward the emergence of resistance to multiple antibiotics is a threat to the management of infections in humans [29]. The fact that some bacteria that cause infections in fish belong to the same genera as bacteria that cause infections in humans is likely to increase the likelihood of spread of antibiotic resistance from aquatic environments to humans [4]. Other studies supporting the multi-resistance recorded in our study have shown that plasmids harboring antibiotic resistance determinants are transferable from fish pathogens and aquatic bacteria, not only to other bacteria of the same genus, but also to *E. coli* and *Salmonella* [40], [84]. *Salmonella* resistance to ciprofloxacin observed in this study is consistent with that of foodborne *Salmonella* worldwide [22]. The results obtained also reveal resistance of Salmonella to other antibiotics, including azithromycin, which are used for the treatment of salmonellosis; however, resistance to azithromycin has already been reported.

The emergence of resistance in microorganisms is a natural process [5]. However, the increasing use of antibiotics and the existence of reservoirs of antibiotic resistance in the environment may greatly accelerate the evolution of multidrug-resistant bacteria [62] and compromise the therapeutic potential of antibiotics. Our findings corroborate the antibiotic resistance observed in other studies [69]. Consequently, it is essential to take into account that the use of antibiotics in fish production impacts on bacterial diversity and promotes bacteria to evolve rapidly not only by mutation and rapid multiplication, but also by DNA transfer [74]. One control strategy is the use of specific antibiotics rather than broad-spectrum antibiotics, in order to avoid affecting beneficial bacteria [63].

## 5 Conclusions

Our study showed that the highest bacterial richness and diversity of Enterobacterales was recorded in the Habascocha lagoon and the lowest richness and diversity in Tipicocha. Sensitivity tests were applied to six bacterial species against 11 antibiotics using the disk diffusion method. *E. coli* was the most resistant bacterium against the antibiotics used. Therefore, is necessary to emphasize that the environmental occurrence of antibiotics and antibiotic resistant bacteria is a global concern. Therefore, it is urgent to undertake practices that minimize the use of antibiotics in fish farms and control the spread of antibiotic resistance. This study also shows that fish farms impact aquatic bacterial diversity and underscores the value of analyzing antibiotic resistance by providing a baseline for future studies and fish farm management in the use of antibiotics. Likewise, the antibiotics authorized for use in Peruvian aquaculture production are amoxicillin, chlorotetracycline, enrofloxacin, florfenicol, flumequine, erythromycin, oxytetracycline, oxolinic acid and sulfonamides. However, there are no records available on the amount of antibiotics used in the fish farms studied. This constitutes an important knowledge gap on antibiotic use and antibiotic-resistant bacterial diversity in inland water fish production. This limitation is particularly acute for some of the most commonly cultured fish species such as *Oncorhynchus mykiss*.

## CRediT authorship contribution statement

**M. Custodio**: Investigation, Conceptualization, Formal analysis, Visualization, Writing – original draft, Writing – review & editing. **R. Peñaloza**: Investigation, Data curation, Visualization, Writing – review & editing. **A. Ordinola**: Investigation, Formal analysis, Visualization, Writing – review & editing. **T. Peralta**: Investigation, Formal analysis, Writing – review & editing. **H. Sánchez**: Investigation, Formal analysis, Writing – review & editing. **E. Vieyra**: Investigation, Formal analysis, Writing – review & editing. **H. De la Cruz**: Investigation, Formal analysis, Writing – review & editing. **J. Alvarado**: Investigation, Visualization, Writing – review & editing.

## Declaration of Competing Interest

The authors declare that they have no known competing financial interests or personal relationships that could have appeared to influence the work reported in this paper.

## Data Availability

Data will be made available on request.
