# Supplementary material for: Diversity of enterobacterales in sediments of lagoons with fish farming activity and analysis of antibiotic resistance
Source: Toxicol Rep. 2023 Feb 11;10:235–44. doi: 10.1016/j.toxrep.2023.02.002 (PMC9950807; doi:10.1016/j.toxrep.2023.02.002)
Supplement: Supplementary file 1 — Supplementary material. [file mmc1.docx]

**Diversity of Enterobacterales in sediments of lagoons with fish farming activity and analysis of antibiotic resistance**

María Custodio^a^*, Richard Peñaloza^a^, Alberto Ordinola-Zapata^b^, Tessy Peralta-Ortiz^b^, Héctor Sánches-Suárez^b^, Enedia Vieyra-Peña^b^, Heidi De la Cruz^a^, Juan Alvarado-Ibáñez^c^

^a^ *Universidad Nacional del Centro del Perú, Facultad de Medicina Humana, Huancayo, Perú*

^b^ *Universidad Nacional de Tumbes, Tumbes, Perú*

^c^ *Universidad Nacional Intercultural “Fabiola Salazar Leguía” de Bagua, Bagua, Perú*

**Supplementary material**

**Table S1.** Characteristics of the evaluated lagoons

| Lagoon | Temperature (°C) | pH | Deep (m) | Area (ha) |
| --- | --- | --- | --- | --- |
| Habascocha | ${9.6}^{1}$ | ${8.04}^{1}$ | ${12}^{3}$ | ${80}^{3}$ |
| Pomacocha | ${9.8}^{1}$ | ${8.06}^{1}$ | $9^{3}$ | ${120}^{3}$ |
| Tranca Grande | ${13.1}^{2}$ | ${7.7}^{2}$ | ${28}^{3}$ | ${164}^{3}$ |
| Tipicocha | ${11.9}^{2}$ | $8^{2}$ | ${10}^{3}$ | ${90}^{3}$ |

^2^Chanamé and Poma (2020), ^2^Chanamé et al. (2020), ^3^Mariano et al. (2011)

**Table S2.** Eigen value, percentage variance and charge value of principal component analysis

|  | PC 1 | PC 2 |
| --- | --- | --- |
| ATM | 0.60712 | -0.020758 |
| GM | 0.33868 | 0.35542 |
| MK | 0.33793 | 0.35812 |
| CAZ | 0.47528 | -0.12603 |
| AMC | 0.15141 | -0.23037 |
| C | 0.2755 | 0.13493 |
| CIP | 0.048275 | 0.39305 |
| CFL | -0.13562 | 0.37945 |
| AZM | -0.18633 | 0.36134 |
| TE | -0.10161 | 0.34638 |
| NA | -0.10966 | 0.32987 |
| Eigenvalue | 3.06 | 2.53 |
| % variance | 28.819 | 21.506 |

**References**

Chanamé, F., Custodio, M., Poma-Chávez, C., & Huamán, A. (2020). Nutrient concentrations and trophic state of three Andean lakes from Junín, Perú. Revista Ambiente e Agua, 15(4), 1–9. https://doi.org/10.4136/1980-993X

Chanamé, F., & Poma, C. (2020). Parámetros limnológicos y productividad piscícola para la piscicultura de trucha arco iris en lagunas altoandinas - Junín, Perú. Prospectiva Universitaria, 17(1), 159–165. https://doi.org/10.26490/uncp.prospectivauniversitaria.2020.17.1400

Mariano, M., Huaman, P., Mayta, E., Montoya, H., & Chanco, M. C. (2011). Contaminación producida por piscicultura intensiva en lagunas andinas de Junín, Perú. Revista Peruana de Biología, 17(1), 137–140. https://doi.org/10.15381/rpb.v17i1.63
